# Morphology of human sweat ducts observed by optical coherence tomography and their frequency of resonance in the terahertz frequency region

**DOI:** 10.1038/srep09071

**Published:** 2015-03-13

**Authors:** Saroj R. Tripathi, Eisuke Miyata, Paul Ben Ishai, Kodo Kawase

**Affiliations:** 1 Nagoya University, Furo-cho, Chikusa Ku, Nagoya 464-8603, Japan; 2 The Hebrew University of Jerusalem, Givat Ram, Jerusalem 91904, Israel; 3 RIKEN, 519-1399 Aramakiaoba, Aoba, Sendai 980-0845, Japan

## Abstract

It is crucial to understand the various biological effects induced by terahertz (THz) electromagnetic waves with the rapid development of electronic and photonic devices operating in the THz frequency region. The presence of sweat glands plays an important role in THz wave interactions with human skin. We investigated the morphological features of sweat ducts using optical coherence tomography (OCT) to further understand such phenomena. We observed remarkable features of the ducts, such as their clear helical structure. The intersubject and intrasubject variations in the diameter of sweat ducts were considerably smaller than the variations in other structural parameters, such as length and number of turns. Based on the sweat duct dimensions and THz dielectric properties of skin measured using terahertz time-domain spectroscopy (THz-TDS), we calculated the resonating frequency of the sweat duct under the assumption of it functioning as a helical antenna. Here, we show that the resonance frequency in the axial mode of operation lies in the THz wave region with a centre frequency of 0.44 ± 0.07 THz. We expect that these findings will further our understanding of the various health consequences of the interaction of THz waves with human beings.

Electromagnetic waves with a frequency extending from a few hundreds of gigahertz (GHz) to a few terahertz (THz) are terahertz waves, which lie between the microwave and infrared regions in the electromagnetic spectrum. Despite its location between these well-developed regions, the THz frequency region remains the least explored due to the lack of efficient emitters and detectors, which can be used in practical applications. However, the development of various optoelectronic technologies, such as femtosecond lasers, semiconductors and crystal growth techniques, has enabled the efficient generation and detection of THz waves at high sensitivity. Applications for this type of radiation have expanded into diverse fields such as homeland security^1^, material characterisation^2^, non-destructive testing and analysis^3^, scientific studies^4^ and information and communication technology^5^. In addition, THz wave applications in biomedicine are of particular interest, and various studies—such as tissue characterisation^6^, cancer detection^7^,^8^, burn assessment^9^, hydration sensing^10^, and blood characterisation^11^—have been reported. Due to such applications of THz radiation, encounters between THz radiation and humans are expected to become common. Therefore, knowledge of the fundamental principles of THz-induced thermal and non-thermal biological effects in human beings is crucial for understanding the health consequences.

The thermal and non-thermal effects of THz waves have been investigated. As THz wave photon energy (1 THz = 4.1 meV) is insufficient to ionise biological molecules, it was assumed that bulk heating of water was mainly responsible for the thermal effects of THz radiation^12^,^13^,^14^,^15^,^16^. However, the high peak electric field THz waves are responsible for various non-thermal biological effects. Exposing human tissue to intense pulses of THz radiation may cause DNA damage^17^ and change gene expression^18^. Other studies on THz-induced non-thermal effects suggest genomic instability^19^, mutagenesis^20^, DNA synthesis^21^, spindle disturbances in cells^22^ and stem cell reprogramming^23^.

Besides such studies on thermal and non-thermal effects induced by THz radiation, recently novel phenomena of skin-THz interaction have been reported. These studies have demonstrated that sweat glands present in the skin play a critical role in THz wave interaction with human beings. It was reported that the sweat ducts act as a low-Q-factor helical antenna due to their helical structure, and resonate in the terahertz frequency range due to their structural parameters, such as helix diameter and helix length^24^,^25^,^26^,^27^. Numerical modelling and experimental data suggest a key role for the sweat duct in characterising the phenomena of resonance behavior^28^. Similarly, it was reported that the presence of the helical-structured sweat duct could play a significant role in millimetre-wave absorption^29^. Moreover, as the sweat duct is controlled by the sympathetic nervous system, it reflects the psychological state of the subject, which allows for remote sensing of mental stress using sub-THz electromagnetic waves^30^. In contrast, some reports have suggested that multiple interference effects play a major role in shaping the spectrum^31^,^32^. The duct acts as a helical antenna based on electromagnetic and antenna theory; the dimensions of the duct and the dielectric properties of the surrounding medium are major factors that determine the resonance frequency^33^. Therefore, sweat duct dimensions, density and distribution as well as the dielectric properties of the stratum corneum are crucial in determining the resonating frequency range, which will enable further insight into THz wave interactions with human beings.

The distribution and density of sweat ducts have been investigated using various methods; these include ductal pore counting^34^, colourimetry^35^ and plastic impression techniques^36^. However, non-invasive visualisation technology—such as optical coherence tomography (OCT)—facilitates morphological investigation of sweat ducts. Several studies have reported non-invasive methods of visualising the human sweat duct using OCT^37^,^38^,^39^. However, few have used OCT to visualise the sweat duct in particular, and investigated the dynamics of the sweat gland and sweat forming mechanism^40^,^41^. Despite implementation of OCT for *in vivo* visualisation of the sweat duct, systematic *in vivo* studies on sweat duct dimensions, distribution, and density remain scarce.

In this study, we explored the morphological features of sweat ducts and their influence on THz-wave-induced effects on the human body. First, we identified the duct's structural parameters and their intersubject and intrasubject variation, and then determined the dielectric properties of sweat ducts to calculate their resonating frequency. We performed OCT on a number of volunteers of different ages and on different regions of the body. We calculated various sweat duct parameters, such as duct length, number of turns in the duct, duct diameter, and thickness of the stratum corneum. We also investigated sweat duct distribution and density on various parts of the body, such as the palms of the hands and soles of the feet. We statistically determined intersubject and intrasubject variation. We used pulsed terahertz time-domain spectroscopy (THz-TDS) to measure the dielectric properties of the skin in the terahertz frequency range. Based on these parameters, we determined the resonance frequency of the human sweat duct.

## Results

### Optical coherence tomography (OCT) of human skin

Human skin consists of three layers—the epidermis, dermis and hypodermis (subcutaneous tissues). The epidermis is the outer layer above which lies a layer known as the stratum corneum, consisting of dead cells known as corneocytes. Underneath the epidermis is the dermis, which contains major skin structures such as sweat glands, nerve endings, blood vessels, sebaceous glands and hair follicles. The innermost subcutaneous fat layer, or hypodermis, bridges the overlying dermis and underlying body constituents. The sweat gland, which lies in the dermis, is connected to the pore (also called acrosyringium) on the outer surface of the skin through the tubular sweat duct. The gland can be arbitrarily divided into the following three major regions^42^, although there are no sharp boundaries: (a) The coiled secretory portion lies in the dermis, (b) the ascending portion of the straight duct that passes through the dermis to the epidermis, and (c) the helical structured portion of the duct from the epidermis that terminates in the stratum corneum. The principal function of eccrine sweat glands is thermoregulation during exposure to a hot environment or during physical exercise^43^,^44^. These glands also become active during emotional sweating induced by pain, anxiety, fear and stress^45^,^46^. In such cases, the sweating rate is under the direct control of the sympathetic nervous system. Thus, large numbers of histological, morphological and physiological studies of the sweat gland have been carried out in the past several decades to understand sweat rate, the perspiration mechanism, and their control by the sympathetic nervous system^47^,^48^,^49^.

We implemented a commercially available swept-source OCT system (Santec Inc. IVS-300) to obtain skin images *in vivo* on different parts of the body, such as the palms of the hands and soles of the feet, to assess the morphological features of sweat ducts. This system consisted of a fibre laser with a centre wavelength of 1310 nm and a scanning rate of 30 KHz. The two-dimensional image data were collected at a rate of 25 frames/s and displayed as a vertical slice in the plane perpendicular to the skin surface. The dimensions of the sweat duct and stratum corneum thickness were determined using the accompanying software (Inner Vision, OCT viewer, Multi Slice viewer, Santec Inc.).

**Figure 1:**
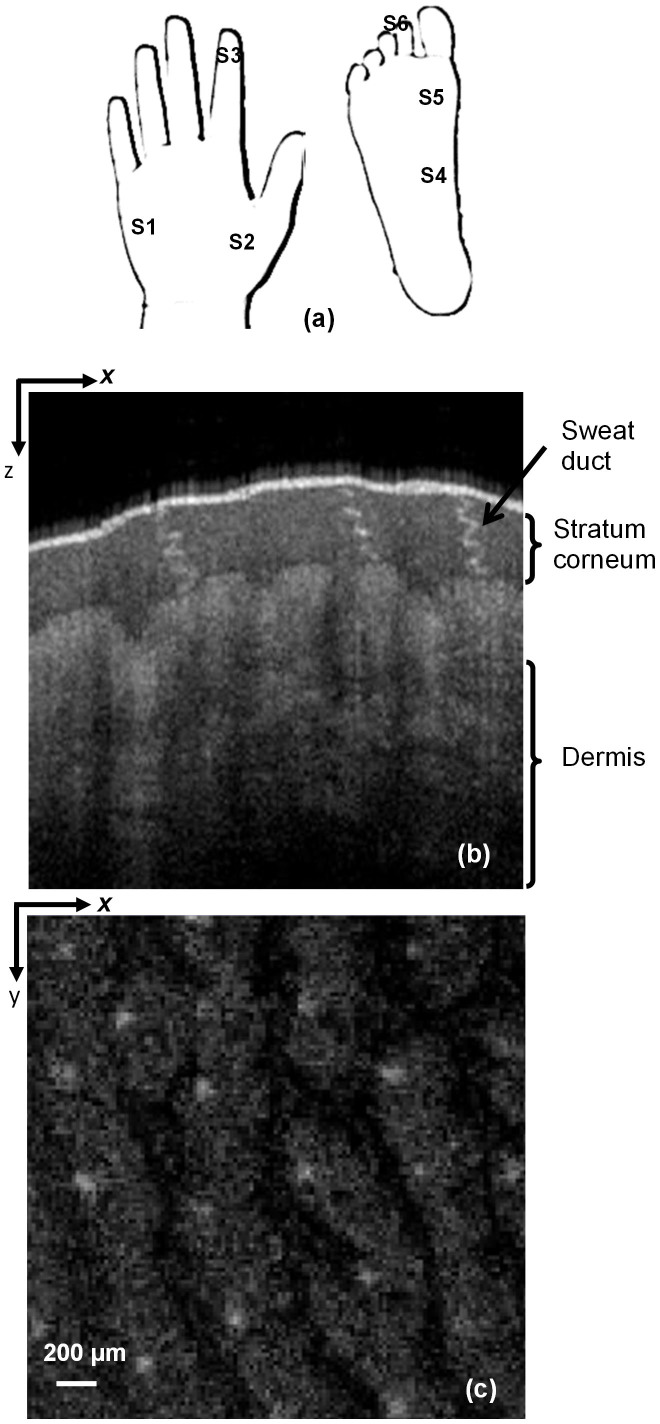
(a) Imaging sites where S1: Base of little finger; S2: Between thumb and wrist; S3: Index finger tip; S4: Inner arch; S5: Mound of big toe; S6: Right second toe (b) Typical optical coherence tomography (OCT) image in the x-z plane showing the stratum corneum, sweat duct and dermis (c) A 2.5 × 2.5-mm en face OCT image in the x-y plane showing the sweat gland duct distribution. Here, the light white spots are the sweat ducts and the light gray features are due to the undulations of the skin surface (fingerprints).

We recruited 32 subjects (12 Male and 10 female with age >10 years and 6 male and 4 female with age <10 years) for the measurements to investigate the intersubject and intrasubject variations in density and parameters of sweat ducts. All subjects or their guardians in the case of minors, gave their informed consent to the research and the research was approved by the University Ethics committee. We performed OCT measurements of each subject *in vivo* at the following locations on the palms of the hands: (a) the base of the little finger (b), between the thumb and wrist, (c) and the right index finger tip. Similarly, we measured (d) the inner arch (e) mound of the big toe, and (f) the right second toe on the foot. Figure 1a shows these measurement sites. A three-dimensional dataset consisting of 255 × 255 × 849 (x, y, z directions) pixels covering a volume of 2.5 × 2.5 × 3.6 mm^3^ was recorded for each measurement. The lateral pixel resolution was 9.8 μm, whereas the depth resolution was 4.2 μm. Here, 125 OCT images were obtained in the x-z plane, and the en face image in the x-y plane was extracted with slice spacing of 4.2 μm. Figure 1b shows a representative image in the x-z plane, in which the spiral lumens of three sweat ducts in the stratum corneum are evident due to the reflection of light. The reflection was due mainly to the large dielectric property mismatch between the keratinous epidermal tissue and the sweat-containing spiral lumen. However, the epidermis and the parts below were not clearly visible due to their high water content. This morphological structure of skin is consistent with previous reports^37^,^39^,^40^. A representative x-y plane image showing a number of sweat ducts arranged in a matrix is shown in Fig. 1c. We used such images to determine the number of ducts per unit area.

### Density and variations in sweat ducts at different measurement sites

**Figure 2:**
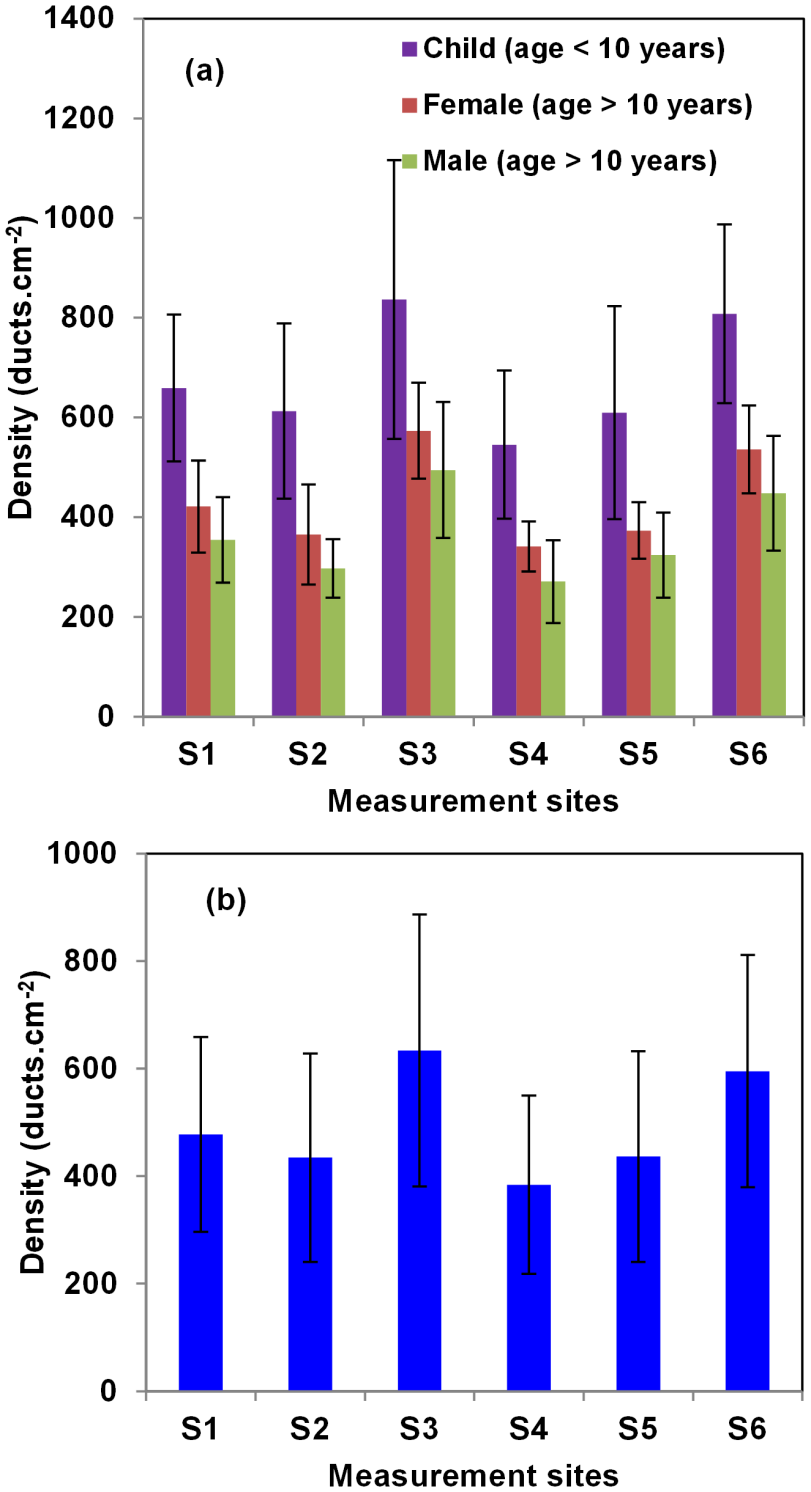
(a) Variation in ensemble averaged duct density at different measurement sites in subjects of different ages and gender. (b) Sweat duct density variation in ensemble averaged over all subjects. In both cases the error bars represents the standard deviation in the sample pool.

The number of sweat ducts within human skin varies from 2–4 million^49^, and ducts are distributed across the body surface. However, for such helical ducts to function as antennas, the number of turns must be higher than three. The majority of such ducts identified by OCT are present on the palms of the hands and feet. Therefore, we determined the duct density mainly in these regions, as shown in Fig. 1a. In our study, we investigated the variation in duct density and other structural parameters for all subjects. Figure 2a shows the duct densities of three different subject groups and surprisingly, the duct densities in all measurement regions were higher in subjects <10 years of age than those in the corresponding measurement regions in adults. Within adults, we observed that the density in female is slightly larger than that of male in all respective measurement regions. Figure 2b shows the average and standard deviation of duct density obtained from Fig.2a. We observed that the duct density was highest at the tip of the index finger (633 ± 252 ducts/cm^2^), whereas measurement site S4 had the lowest (384 ± 165 ducts/cm^2^) duct density. Notably, the standard deviations of duct density at all sites were relatively high. This was due mainly to collection of data from subjects of different ages. We determined duct density in subjects <10 years of age and the high duct density is due to the fact that the glands start to appear after a few gestational weeks, their development is complete after 22 weeks of gestation, and the number remains fairly constant^49^.

### Duct parameter variation

We examined the variations in sweat duct diameter, duct length and number of turns. We also measured the dielectric properties of the stratum corneum using pulsed THz-TDS in reflection mode, which required accurate determination of stratum corneum thickness. Therefore, the thickness of the stratum corneum and its intersubject and intrasubject variations were investigated. We first investigated the sweat duct parameters in one measurement region to acquire information about the variation in a particular area of a single subject (site S3: index fingertip). The percentage variation in all parameters was small (for further details, see Supplementary Materials). Therefore, we evaluated the abovementioned parameters of a few representative ducts from one region.

**Figure 3:**
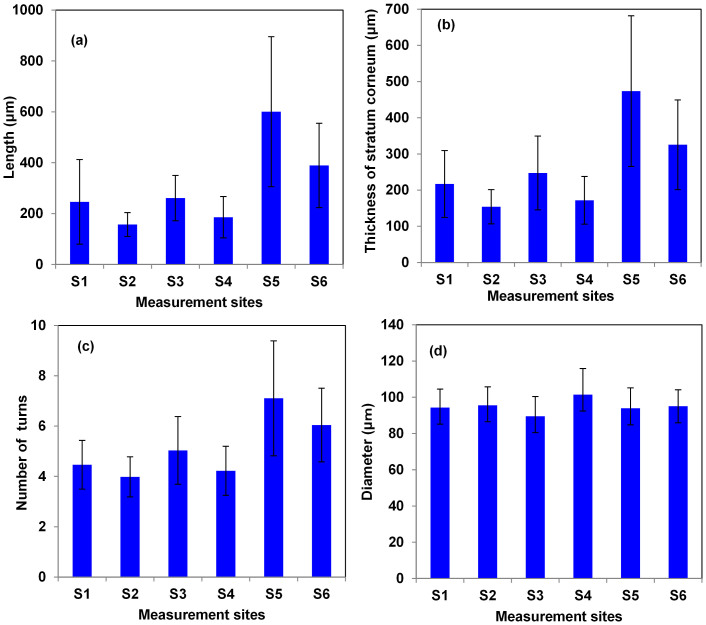
(a) Lengths of the ducts and their standard deviations. (b) Thickness of the stratum corneum and their standard deviation. (c) Number of turns and their standard deviations (d) Diameter of the sweat ducts and their standard deviation

Figure 3a shows the lengths of the sweat ducts in different measurement regions. Duct length varied widely among the measurement regions. The longest ducts were found in the S5 region (average length, 600 ± 294 μm), whereas the shortest ducts were in region S2 (157 ± 46 μm). However, the relative standard deviation (RSD), which is calculated as: (standard deviation/mean ×100), was higher in the case of measurement region S1 (RSD = 67%), whereas measurement region S2 had the lowest value (29%). Similarly, Fig. 3b shows that the length of the sweat ducts was proportional to the stratum corneum thickness at all measurement sites. The RSD in region S5 was highest (43%) compared with that in other areas. This was due mainly to variation in stratum corneum thicknesses among the subjects. In general, subjects who walk a lot or play tend to have a thicker stratum corneum, and the length of sweat ducts increase in proportion to the stratum corneum thickness. However, in some cases, the duct length was longer than the thickness of the stratum corneum, due to the presence of tilted ducts. As the number of turns is important for a duct to function as a helical antenna, we determined the number of turns and the variation thereof, as shown in Fig. 3c. The number of turns in the sweat ducts was highest in region S5, and that in region S2 the fewest.

We computed Pearson's correlation coefficients among stratum corneum thickness, duct length, and number of turns. There was a very high correlation coefficient (0.99) between stratum corneum thickness and duct length, indicating that the ducts extended throughout the stratum corneum. Similarly, the correlation coefficient between duct length and number of turns was 0.98. This indicates that duct length is proportional to the number of turns, which results in sweat ducts having an almost constant pitch angle. This facilitates function of the sweat duct as a helical antenna in the THz frequency region.

Figure 3d shows the variations in sweat duct diameters in different regions. Unlike the other characteristics, the sweat ducts had similar diameters, regardless of measurement location. The RSD (14%) was highest in region S4, and the lowest in region S6 (9%). The highest (101 ± 14 μm) and lowest (89 ± 10 μm) duct diameters were in regions S4 and S3, respectively. The average duct diameter was 95 ± 11 μm.

### Calculating the resonance frequency of a sweat duct

Sweat ducts might function as low-Q helical antennas, the resonance frequency of which is dependent on the structural parameters of the ducts. The helix is principally characterized by its length (*L*), diameter (*D*) and the number of turns in the helix (*N*). Based upon these parameters, the other parameters such as its circumference, pitch angles and total length can be calculated (refer supplementary materials for further details). According to classic electromagnetic theory, a helical antenna resonates in two different modes: (a) Normal or broadside mode, in which the directivity of the antenna lies normal to the axis of helix, and (b) axial mode (also known as end-fire mode) in which the directivity of the antenna lies along the axis of the helix. Polarisation of the wave is circular with the axial mode of radiation. As sweat ducts are arranged in the skin in such a way that the helix axis is perpendicular to the skin plane, the axial mode of operation is expected to be dominant. Moreover, the diameter and spacing of the duct must be large fractions of the wavelength to excite the axial mode^33^. The other conditions that must be satisfied for a duct to act as a helical antenna are number of turn (N) > 3 and 12° < pitch angle (α) < 14°. In our measurements, the average number of duct turns was 5 and the pitch angle was ~12°, satisfying the resonance conditions (Refer to Supplementary Materials for further details). Following the lead of Feldman et al.^24^,^25^,^26^,^27^ the sweat duct is considered as a pipe filled with water. Proton conductivity, enhanced by the large lipid/water interface in the duct was proposed as the source ac conductivity fast enough to react to the incoming electromagnetic wave^25^. Under these conditions the duct can be abstracted to an ideal helix embedded in a dielectric medium. Consequently, the resonance frequency is governed by the dielectric properties of the medium. With the consideration of dielectric properties of stratum corneum, the region of frequency of resonance in axial mode of operation is obtained using the following expression^33^:
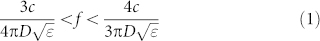
where, *c* is the velocity of light, *D* is the helix diameter, and *ε* is the dielectric constant of the stratum corneum. The resonance frequency can be written so as to obtain circular polarisation under optimised conditions:
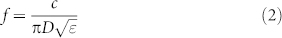
Here, it is important to note that not only sweat diameter duct but also the stratum corneum dielectric constant plays a critical role determining sweat duct resonance frequency. Therefore, we used THz-TDS to determine accurately the dielectric property values of the stratum corneum.

### THz dielectric properties of the stratum corneum as measured by THz TDS

**Figure 4:**
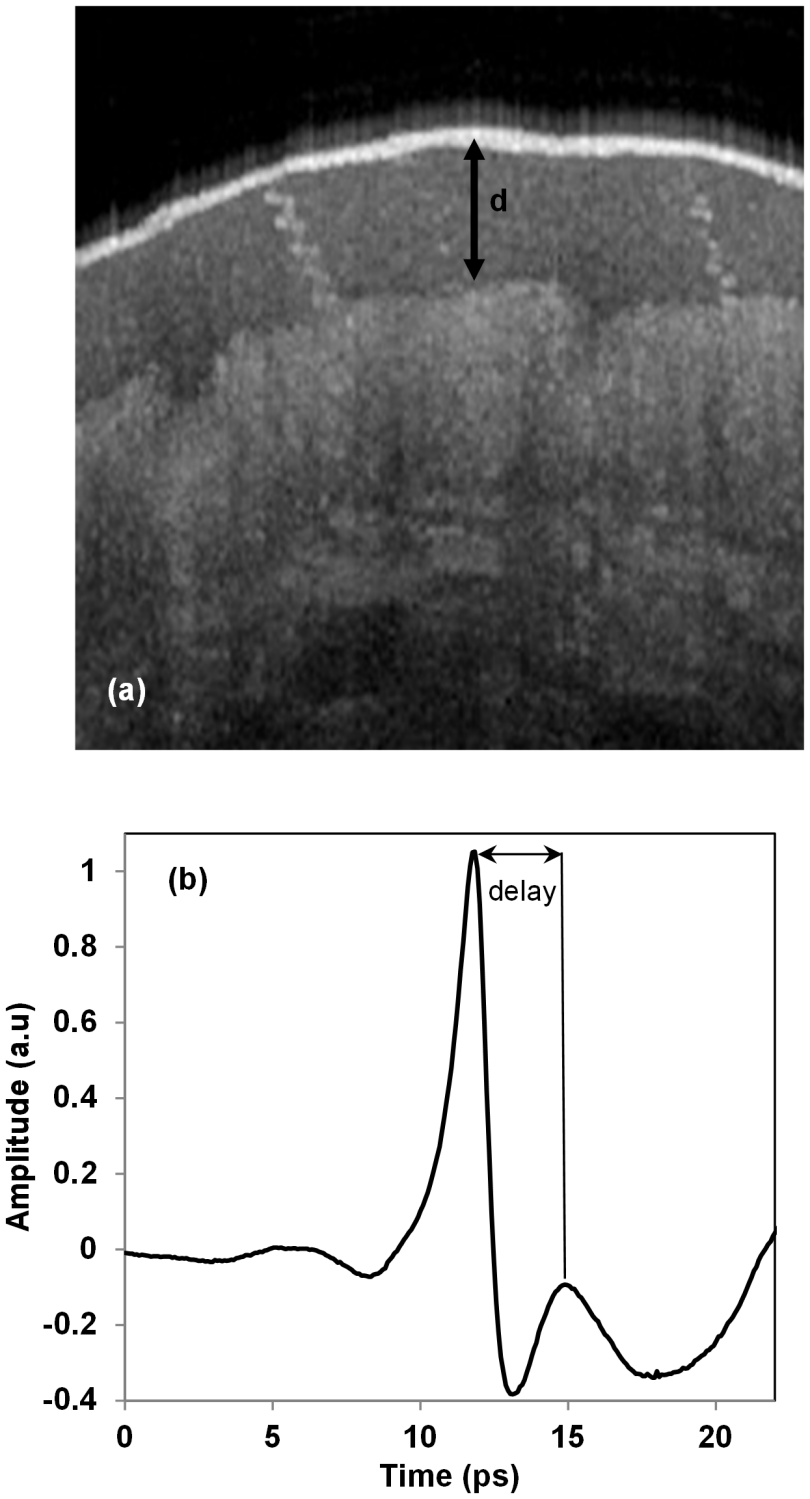
(a) Thickness (d) of the stratum corneum. (b) THz time-domain pulse reflected from the palm of the hand.

THz TDS can determine the complex refractive index of a material by probing it with a sub-picosecond pulsed THz wave. The THz pulse is either transmitted through or reflected back from the sample under investigation to obtain spectroscopic information. In this study, we developed THz TDS using reflection geometry, in which the pulsed THz wave was incident upon the palm of the hand. As the penetrability of THz waves is high in relatively moisture-free samples, THz TDS facilitates determination of the refractive index of the stratum corneum. However, the total path length travelled by THz waves in the medium is an essential value for determination of the refractive index. Therefore, we used OCT to measure stratum corneum thickness. Figure 4a shows a representative OCT images of the stratum corneum, sweat ducts, and epidermis. The thickness of the stratum corneum was determined directly using the software that accompanied the OCT setup. After each OCT measurement and stratum corneum thickness determination, we immediately performed a THz wave measurement and obtained the refractive index of the stratum corneum.

Figure 4b shows a representative THz pulse reflected from the boundary between the windows material and the stratum corneum. Another pulse, followed by the first main pulse, was reflected from the boundary between the stratum corneum and the epidermis. Based on the time delay and stratum corneum thickness (d) obtained by OCT, we determined the dielectric constant to be 5.1 ± 1.3. The variation in the dielectric constant is due mainly to the different levels of moisture in the subjects. The measured values showed considerable consistency with those reported previously^50^,^51^. Finally, using Eq. 2 and considering the duct diameter, the dielectric constant and their variations, we determined that the centre resonance frequency was 442 ± 76 GHz. However, the frequency range obtained from Eq. 1 was 332–590 GHz. It is worth noting that circular dichroism, a signature of the helical antenna, was detected in skin reflectance measurements at 380 GHz^26^.

## Conclusion

Recent studies investigating the bioeffects of THz have demonstrated that THz waves interact with human skin in various ways. In this study, we investigated the morphological structure of the sweat duct and calculated the resonance frequency to further understand the THz wave–skin interaction. We found for the first time that sweat duct diameter is consistent regardless of the measurement site or subject. Moreover, we determined the THz dielectric properties of human skin and based upon these values, we numerically determined the resonating frequency of helical ducts, which was 442 ± 76 GHz. We believe that these findings will facilitate further investigation of the THz-skin interaction and provide guidelines for safety levels with respect to human exposure to electromagnetic waves at these frequencies. Moreover, physiologists, dermatologists and medical practitioners can take advantage of this knowledge to predict sweating rates and other mechanisms associated with sweat duct dimensions and distribution.

## Methods

### Terahertz time-domain spectroscopy

**Figure 5:**
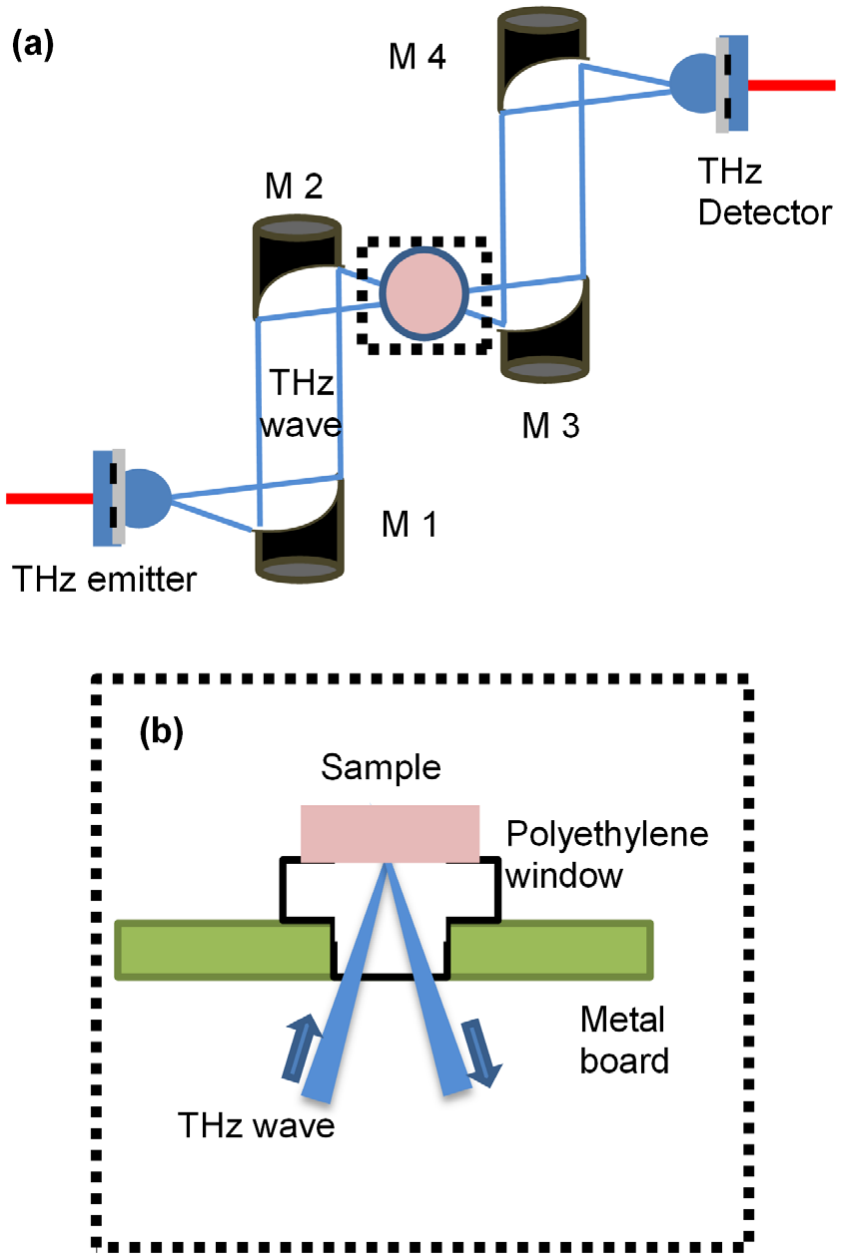
(a) A schematic of the experimental setup for the THz wave reflection measurements. (b) Side view of the dotted box in Fig. 5a.

We developed a THz time-domain spectrometer with a reflection mode (Fig. 5a), in which the emission and detection of the THz time domain pulses were accomplished using a low-temperature-grown gallium arsenide photoconductive antenna. We used a femtosecond fibre laser (Femtolite IMRA., pulse width <100 fs, average power ~20 mW) to pump and probe the emitter and detector antenna, respectively. We used a bow-tie–type antenna as an emitter, which emit waves in the low THz frequency region. The emitted THz wave was collimated by mirror M1 and then focused onto the sample using mirror M2. The reflected signal from the sample was then again collimated and focused onto a detector using mirrors M3 and M4, respectively. The palm was supported using a metallic board with a hole, as shown in Fig. 5b. We inserted a polyethylene (PE) plug in the hole as window material. PE has a low refractive index and low THz absorption and is used widely as a window material in THz measurement systems.

## Author Contributions

Development of terahertz time domain spectrometer, dielectric properties measurements of skin, and OCT measurements were done by S.R.T. and K.M. The data were analysed by S.R.T. and P.B.I. The manuscript text and the figures were written by S.R.T. This project was conceived and supervised by K.K. All authors have discussed and reviewed the manuscript.

## Supplementary Material

Supplementary Information Supplementary materials
