# Supplementary material for: Morphology of human sweat ducts observed by optical coherence tomography and their frequency of resonance in the terahertz frequency region
Source: Sci Rep. 2015 Mar 13;5:9071. doi: 10.1038/srep09071 (PMC4357862; doi:10.1038/srep09071)
Supplement: Supplementary Information — Supplementary materials [file srep09071-s1.pdf]

Morphology of human sweat ducts observed by optical coherence tomography and their frequency of resonance in the terahertz frequency region

Saroj R. Tripathi[1*], Eisuke Miyata[1], Paul Ben Ishai[2] and Kodo Kawase[1,3]

[1]Nagoya University, Furo-cho, Chikusa Ku, Nagoya 464-8603, Japan

[2]The Hebrew University of Jerusalem, Givat Ram, Jerusalem 91904, Israel

[3]RIKEN, 519-1399 Aramakiaoba, Aoba, Sendai 980-0845, Japan

[*]Corresponding author: tripathi@nuee.nagoya-u.ac.jp

Variation in duct structural parameters within a single measurement region:

We used the OCT image of the tip of the right index finger (male, 26 yr) to investigate intra-region variation (one region of one subject). We labelled the ducts A, B, C, D….. O. Figure S1 shows the variation in duct diameter, the RSD value was 8%. Similarly, the variations in duct length and the number of turns in each duct are shown in Figs. S2 and S3. RSDs of 6% and 14% were observed, respectively. We assessed the characteristics of a few representative ducts to avoid the need for a complex calculation.

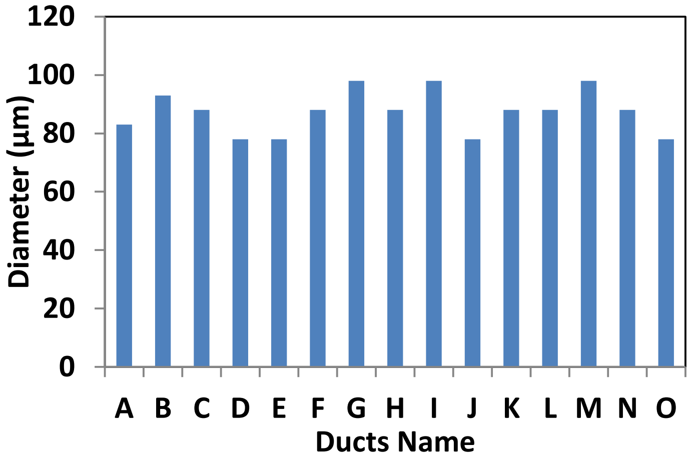

Figure S1: Variation in duct diameter.

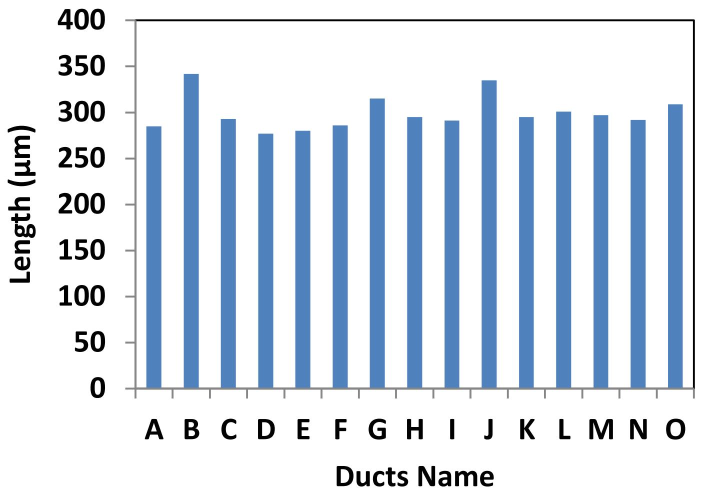

Figure S2: Variation in duct length.

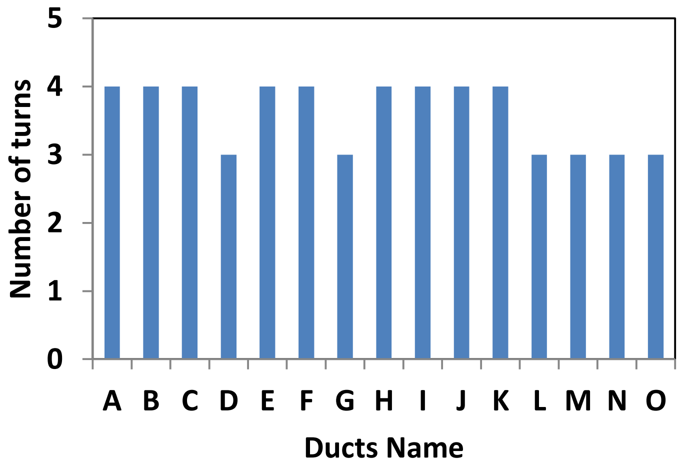

Figure S3: Variation in the number of turns.

Helical antenna properties:

Helix geometry is defined by the following structural parameters.

Length of the helix = L

Diameter of the helix = D

Circumference of the helix (C) = πD

No. of turns in the helix = N

Spacing between the turns (S) = L/N

Pitch angle (α) = $\tan^{-1}(S/\pi D)$

Length of the wire between each turn (L_0) = $\sqrt{(S^2+C^2)}$

Total helix length (L_n) = $N \times L_0$

We obtained an average duct diameter, D = 95 μm, no. of turns = 5 and helix length = 306 μm from our OCT measurements. Using the formula above, we computed spacing $S \approx 61$ μm, pitch angle $\alpha \approx 12°$, length of the wire between each turn $L_0 \approx 304$ μm and total helix length $L_n \approx 1.52$ mm.

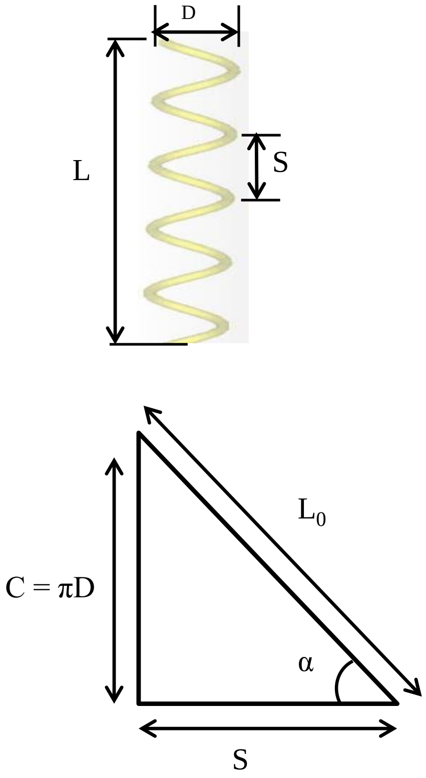

Figure S4: Structural parameters of helix.
